# MCPIP1, alias Regnase-1 binds and cleaves mRNA of C/EBPβ

**DOI:** 10.1371/journal.pone.0174381

**Published:** 2017-03-22

**Authors:** Barbara Lipert, Mateusz Wilamowski, Andrzej Gorecki, Jolanta Jura

**Affiliations:** 1 Jagiellonian University, Faculty of Biochemistry, Biophysics and Biotechnology, Department of General Biochemistry, Krakow, Poland; 2 Jagiellonian University, Faculty of Biochemistry, Biophysics and Biotechnology, Department of Physical Biochemistry, Krakow, Poland; Korea University, REPUBLIC OF KOREA

## Abstract

CCAAT/enhancer-binding protein beta (C/EBPβ) is a transcription factor controlling a broad range of genes essential for homeostasis, including genes related to immune functions, inflammation, metabolism and growth. Monocyte chemoattractant protein-1-induced protein 1 (MCPIP1) also called as Regnase-1 is an RNase and has been shown to decrease the stability of short-lived transcripts coding for inflammation-related proteins, including IL-1β, IL-6, IL-2, IL-8, IL-12b, IER-3, c-Rel. We found previously that the half-life of the C/EBPβ transcript is regulated by MCPIP. To understand the mechanism driving down-regulation of C/EBPβ by MCPIP1, we applied an *in vitro* cleavage assay, followed by a luciferase-reporter assay and RNA immunoprecipitation (RIP). We demonstrated that MCPIP1 recognizes regions of the 3’UTR of C/EBPβ mRNA and promotes its decay by introducing direct endonucleolytic cleavage.

## Introduction

Modulation of mRNA stability has been found to be responsible for 40–50% of all changes in gene expression [1,2]. Research in this field has revealed the importance of *cis*-acting elements located predominantly in the 3’ untranslated region of an mRNA (3’UTR). These elements enable the interaction of mRNA transcripts with an mRNA-decay complex where unspecific nucleases promote degradation. Since the degradation involves 5’-to-3’ and/or 3’-to-5’ exonucleases, their attack must be preceded by a hydrolysis of the 7-metylo-guanine cap on the 5’ end and/or removal of a poly(A)-tail on the 3’ end of an mRNA [3]. However, an efficient way for instantaneous exposition of both mRNA ends for degradation is an intramolecular cleavage by endonucleases. A recently discovered endonuclease capable of mRNA cleavage is known as MCPIP1. This protein is essential for the degradation of short-lived transcripts coding for inflammation-related proteins, including IL-1β, IL-6, IL-2, IL-8, IL-12b, IER-3, c-Rel [4–8]. It has been shown that MCPIP1 binding of an mRNA depends on a conserved stem-loop structure in the 3’UTR. The ribonucleolytic activity of MCPIP1 has been attributed to a PIN (PilT N terminus) like domain, where four Asp residues (D141, D225, D226, D244) in the catalytic center determine RNase activity [9]. Interestingly, the PIN-like domains of two MCPIP1 molecules have been shown to interact with each other and this seems to be critical for MCPIP1 activity *in vitro* [10].

In this study, we aimed to analyze how MCPIP1 regulates the stability of mRNA coding for C/EBPβ (CCAAT/enhancer-binding protein beta). C/EBPβ is a bZIP (basic leucin zipper) transcription factor and belongs to a larger family of C/EBP proteins. C/EBP-binding motifs have been found in the promoters of various genes, including genes encoding the inflammatory cytokines IL-6, IL-1β, TNF-α, IL-8 and IL-12b [11–16]. Our previous work has shown that MCPIP1 decreases the half-life of mRNA coding for C/EBPβ in HepG2 cells, and that this effect requires the presence of PIN domain [17]. Here, we demonstrate that MCPIP1 degrades mRNA coding for C/EBPβ. We indicate regions of its 3’UTR that are recognized and directly cleaved by MCPIP1 *in vitro*. Our observations are further confirmed in HepG2 cells by a luciferase-reporter assay and RNA immunoprecipitation (RIP).

## Materials and methods

### Luciferase assay

Fragments of the 3’UTR C/EBPβ were amplified with PCR using a specific primers (Table 1) to amplify *C/EBPβ* sequence (accession number: NM_005194.3). The last nucleotide of the STOP codon of C/EBPβ mRNA in this paper was numbered “0”. Amplified 3’UTR C/EBPβ fragments were cloned to pmir-GLO plasmid (FJ376737; Promega) at XhoI and SalI restriction sites and sequenced. The PCR product of *MCPIP1* (accession number: NM_025079) generated by PCR with 5’ atggatccgagtctgagctatgagtgg 3’ and 5’ ggaattccctcactggggtgctgg 3’ primers was inserted into the pc-DNA3.1 vector (Invitrogen) at BamHI and EcoRI restriction sites. HepG2 cells were co-transfected with luciferase reporter plasmid pmir-GLO containing the whole 3’UTR of C/EBPβ or its fragments and expression vector pc-DNA3.1 coding for a wild-type MCPIP1 or its mutant MCPIP1-D141N. Cells were co-transfected with an empty pmir-GLO and pcDNA3.1vectors served as an external reference. After 24 h of incubation, cells were lysed and supernatants were subjected for analysis performed with the Dual-Luciferase Reporter Assay System (E1910; Promega).

**Table 1:** Primers used for amplification of 3’UTR C/EBPβ mRNA.

3’UTR of C/EBPβ mRNA	Forward primer 5’-3’	Reverse primer 5’-3’
**-60-624**	ttactcgagctgcggaacttgttcaagcag	attgtcgactttttttactgcccccaaaaggc
**15–624**	attctcgagcgtccccctgccggcc	attgtcgactttttttactgcccccaaaaggc
**15–270**	attctcgagcgtccccctgccggcc	attgtcgacggcagagggagaagcagaga
**268–450**	attctcgagtctctgcttctccctctgcc	attgtcgaccatcaacagcaacaagcc
**432–624**	attctcgagggcttgttgctgttgatg	attgtcgactttttttactgcccccaaaaggc

Primers sequence design was done on the basis of cDNA (NM_005194.3) sequence.

### Protein expression and purification

Full length human MCPIP1 (WT) and MCPIP1 (D141N) recombinant proteins were produced according to procedures previously described [18]. Two additional constructs expressing PIN domain: MCPIP1 PIN and MCPIP1 PIN-D141N, spanning 131–327 residues, were generated by PCR with the following primers: 5’ atggatccgatctgcgcccggtggttatc 3’ and 5’ tactcgagttagctcggacgttccgggtggaa 3’, then cloned to BamHI and XhoI restriction site in modified pET-21a expression vector containing a His-tag at the N-terminus. MCPIP1 PIN and MCPIP1 PIN-D141N were purified by Ni^2+^ affinity chromatography, followed by size exclusion chromatography.

### *In vitro* mRNAs synthesis and purification

Human C/EBPβ transcripts were amplified by PCR on the basis of the *C/EBPβ* sequence: NM_005194.3. PCR products were inserted into a pcDNA3.0 vector (Invitrogen) at HindIII and EcoRI restriction sites. To obtain 3’UTR C/EBPβ mRNA (-3–19) synthetized oligonucleotides 5’ ttaaagctttagcgcggcccccgcgcgcgtcgaattctta 3’ and 5’ taagaattcgacgcgcgcgggggccgcgctaaagctttaa 3’ were denatured then annealed to achieve dsDNA template, followed by phosphorylation with T4 polynucleotide kinase. Next, the template was ligated to pcDNA3.0 vector at the blunt end DraI restriction site. C/EBPβ transcript fragments were synthetized using a T7 RNA polymerase kit (TranscriptAid T7 high Yield Transcription Kit; ThermoFisher) according to the manufacturer’s procedures. Prior mRNA synthesis templates were linearized at the EcoRV site. Synthetized transcripts were purified using a phenol-chloroform extraction procedure [19]. Next, C/EBPβ transcripts were precipitated overnight in absolute ethanol at -20°C. After precipitation, transcripts were dissolved in RNAse free water. RNA concentration was determined using a NanoDrop 1000 spectrophotometer (Thermo Scientific). Purified transcripts were stored at -80°C. Before conducting experiments, transcripts were thawed and then denatured at 90°C for 5 min.

### mRNAs cleavage assay

Purified recombinant MCPIP1 or MCPIP1 PIN was incubated with selected 3’UTR C/EBPβ transcripts. Cleavage reactions were conducted at 37°C in a buffer (50 mM Tris, 150 mM NaCl, 2.5 mM MgCl2, 2.5 mM DTT, 0.5 mM EDTA, 0.025 mM ZnCl2, pH 8.3). Samples were prepared at 20 μl in molar ratio: 1 pmol RNA to 5 pmol MCPIP1. Samples were collected at selected time points and frozen to stop the reaction. Cleaved transcripts were visualized through 4% denaturing polyacrylamide gel electrophoresis in TBE buffer. Before loading on the gel, samples were mixed with RNA Gel Loading Dye (#R0641 ThermoFisher) and denatured for 15 minutes at 70°C. Electrophoresis was performed at 80V, then the gel was stained with SimpleSafe (EuRX) and visualized using a ChemiDoc MP imaging system (BioRad).

### Electrophoretic Mobility Shift Assay (EMSA)

Purified full length MCPIP1-D141N or protein fragment containing mutant form of PIN domain, MCPIP1 PIN-D141N were incubated with selected 3’UTR C/EBPβ transcripts. Reactions were performed at 37°C in a buffer (25 mM Tris, 150 mM NaCl, 2.5 mM MgCl2, 2.5 mM DTT, 0.5 mM EDTA, 0,025 mM ZnCl2, pH 8.3). Samples were mixed using constant 2 pmol amount of RNA (RNA: protein molar ratio were used as follows: 1:0; 1:2.5; 1:12.5; 1:25; 1:37.5; 1:50) and were incubated on ice for 20 minutes. Subsequently, samples were loaded on 0.8% agarose gel that was pre-stained with SimpleSafe (EuRX) dye. Electrophoresis was carried out in TAE buffer at 65 V.

### Cross-linked RNA immunoprecipitation

Human hepatocellular liver carcinoma cell line (HepG2) was purchased from ATCC. Cells were grown in a humidified atmosphere at 5% CO_2_ in DMEM containing 1 g/l of glucose (Lonza, Switzerland) and supplemented with 5% FBS (Lonza, Switzerland). For the experiments, cells on passages 100–110 were seeded on the poly-L-lysine-coated cell culture plates (BD Falcon, USA). PCR products of MCPIP1 generated by 5’ atgcggccgcagctatgagtggc 3’ and 5’ ttaggatccacaggcagcttactcactg 3’ were inserted into a pFLAG-CMV-2 vector at NotI and BamHI restriction sites. Cells were transfected with MCPIP1 pFLAG-CMV-2 expression vector or empty pFLAG-CMV-2 vector (E7033; SIGMA). To perform crosslinking, cells on the plates were treated with formaldehyde (432173111; POCh, Poland) in a final concentration of 1% for 10 minutes. Cells were collected from plates and washed twice using cold PBS buffer with the addition of a protease inhibitors cocktail (P8340, Sigma-Aldrich). Cells were centrifuged at 120 RCF for 10 minutes at room temperature and lysed in a buffer containing 50 mM HEPES, 400 mM NaCl, 1 mM EDTA, 1 mM DTT, 10% glycerol, 0.5% Triton X-100 (pH 7.5), supplemented with an RNase inhibitor (037–1000; A&A Biotechnology) and protease inhibitor cocktail (P8340, Sigma-Aldrich). The pellet was re-suspended in the lysis buffer, frozen in liquid nitrogen and then thawed rapidly to promote cell disruption. Subsequent immunoprecipitation of MCPIP1 complexes with RNA was done in IP buffer: 50 mM HEPES, 150 mM NaCl, 1 mM EDTA, 2 mM DTT, 10% glycerol, 0.5% Triton X-100 (pH 7.5) supplemented with an RNase inhibitor and protease inhibitor cocktail. Samples were incubated overnight with 50 μl Protein A agarose (11719408001; Roche) and 30 μg monoclonal anti-FLAG M2 antibody 1:1000 (F1804; SIGMA). Next, samples were washed five times in 1 ml IP buffer, followed by centrifugation at 300 RCF for 2 minutes. For proteinase K treatment (V302B, Promega), 50 μg of enzyme was added to samples. Reverse of cross-linking was done in RIP buffer: 50 mM HEPES, 100 mM NaCl, 5 mM EDTA, 10 mM DTT, 10% glycerol, 1,5% SDS, 0.5% Triton X-100 (pH 7.5) supplemented with RNase inhibitor. Samples were incubated in RIP buffer at 70°C for 1 hour, then supernatants were collected for further analysis. All of the above buffers were made using DEPC treated water. Transcripts were extracted using a phenol-chloroform procedure [19]. Before RNA precipitation in isopropanol, Vivid Violet was added to samples as a co-precipitating factor (E4502-01; EURx). Precipitation was performed at -20°C for 2 hours. Purified RNA was reverse transcribed to cDNA using random hexamer primers (E0101-01; EURx) and MMLV reverse transcriptase (M1701; Promega). Real time PCR analysis of mRNA was done using Real-Time 2xPCR Master Mix (2005–100; A&A Biotechnology) and the Eco Real-Time PCR System (Illumina). The following primers were used: C/EBPβ 5’ agcgacgagtacaagatccg 3’ and 5’ gctgctccaccttcttctgc 3’, actin 5’ caagagatggccacggctgctt 3’ and 5’ caggtctttgcggatgtccacg 3’, 28S RNA 5’ cacccactaatagggaacgtg 3’ and 5’ ctgacttagaggcgttcagtc 3’.

### Western blotting

For western blotting analysis the following antibodies were used: monoclonal anti-FLAG M2 antibody 1:1000 (F1804; SIGMA) and peroxidase-conjugated goat anti-mouse antibody 1:10000 (554002; BD Pharmingen). Immobilon transfer membrane (IPVH00010; Millipore) was incubated with Immobilon Western Chemiluminescent HRP Substrate (WBKLS0500; Millipore) and visualized using the ChemiDoc MP imaging system (BioRad).

## Results

### MCPIP1 recognizes 3’UTR of C/EBPβ mRNA

**Fig 1:**
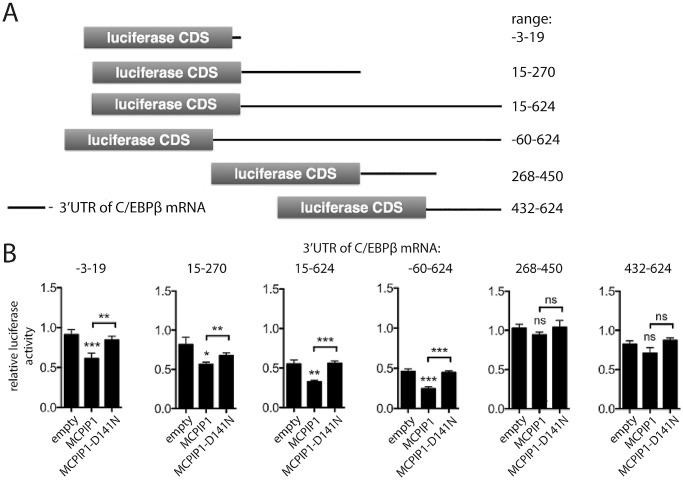
Identification of MCPIP1 target regions within the 3'UTR of C/EBPβ mRNA. **A**. Schematic representation of genetic constructs used in luciferase activity assays. **B**. Luciferase assay of HepG2 cells transfected with a pmir-GLO reporter vector carrying a relevant fragment of the 3'UTR of C/EBPβ mRNA linked to a coding sequence of firefly luciferase and pcDNA3.1 expression vector coding for wild type MCPIP1 or MCPIP1 with abolished RNase activity (MCPIP1-D141N). Empty pcDNA3.1 vector served as a control. All values were normalized to the same external reference i.e. value measured for cells co-transfected with empty luciferase-expressing vector and an empty pcDNA3.1 expression vector. Graph shows mean ± SD n = 3; *p<0.05, **p <0.02 ***p<0.001, ns—not significant (Student-t test).

To specify which 3’UTR region of the C/EBPβ mRNA is involved in the MCPIP1-driven down-regulation, we prepared a set of reporter vectors harboring different sections of the 3’UTR of C/EBPβ mRNA (Fig 1A) which were linked to a luciferase coding sequence (CDS). Constructs were introduced to HepG2 cells together with plasmids coding for MCPIP1 or MCPIP1 lacking RNase activity (MCPIP1-D141N). Luciferase assay result showed, that cells expressing luciferase cDNA with the full-length 3’UTR (-60-624) exibit lower activity of luciferase in the presence of MCPIP1, but not MCPIP1-D141N (Fig 1B). Amongst 3’UTR fragments, the inhibitory effect of MCPIP1 was observed to have similar activity for the regions spanning nucleotides 15 to 624 and 15 to 270 as well as for a short region near the STOP codon (-3 to 19 nt). Mutant form of MCPIP1 (MCPIP1-D141N) had no effect on luciferase activity, thus, we concluded that these regions are important in the regulation of C/EBPβ mRNA stability controlled by the active form of MCPIP1. Only distant regions of the 3'UTR of C/EBPβ mRNA (268 to 624 nt) remained resistant to MCPIP1 activity. Interestingly, a comparison between reference cells (transfected with an empty plasmids) revealed that the 3’UTR of C/EBPβ mRNA strongly influenced luciferase activity, also irrespectively of MCPIP1. This implies that the 3’UTR of C/EBPβ mRNA contains other MCPIP1-independent regulatory elements. The strongest inhibition of luciferase activity was observed for the full-length of the 3’UTR of C/EBPβ mRNA. The regulatory motifs are located in the first (15–270) and last (432–624) part of the 3’UTR and their effect on mRNA processing seems to be additive.

### MCPIP1 binds the 3’UTR of C/EBPβ mRNA

**Fig 2:**
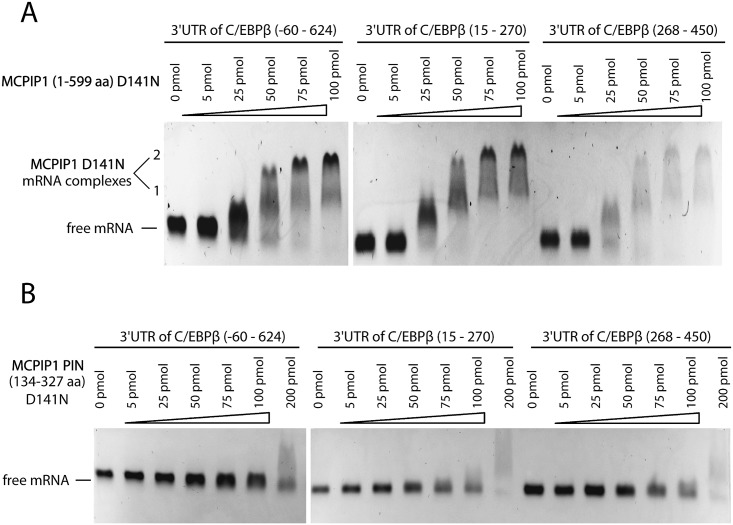
MCPIP1-D141N with completely abolished RNase activity retains interaction ability against the 3'UTR of C/EBPβ mRNA. Results of electrophoretic mobility shift assays performed with increasing amounts (0–200 pmol) of recombinant MCPIP1-D141N **A**. or PIN domain alone, MCPIP1 PIN-D141N **B**. incubated with 2 pmol of *in vitro*–transcribed the 3'UTR fragments of C/EBPβ mRNA.

At this point it was not clear if the loss of luciferase activity results from direct or indirect effects of MCPIP1 on mRNA processing. For example, MCPIP1 might degrade the transcript coding for the C/EBPβ mRNA regulator. Thus, in the next step of our study we checked if MCPIP1 can interact directly with the 3'UTR of C/EBPβ mRNA. To achieve this objective we performed electrophoretic mobility shift assays (EMSA). Additionally, to identify the functional region we analyzed a set of fragments of the 3'UTR of C/EBPβ mRNA. RNA was incubated with enzymatically inactive mutant of recombinant MCPIP1 (MCPIP1-D141N) to prevent degradation. We found that MCPIP1-D141N efficiently binds all analyzed 3’UTR fragments, including the distant region (ranged 268–450 nt) (Fig 2A).

We observed that an increased amount of protein leads to the formation of heavier complexes. We concluded that these are oligomeric forms of MCPIP1 and thus a homo-oligomer is a functional form of MCPIP1. We analyzed the interaction between the 3'UTR fragments of C/EBPβ mRNA and the PIN domain of MCPIP1 harboring the point mutation, D141N. We did not observe the oligomerization of recombinant PIN domain with mutation of one conserved amino acid residue: MCPIP1 PIN-D141N, even at the highest quantity of the RNA (200 pmol). This clearly shows that MCPIP1 needs other domains to create oligomers and that the PIN domain alone is not sufficient to generate such complexes. Furthermore, MCPIP1 PIN-D141N domain showed weak potential to bind any of the analyzed the 3'UTR fragments of C/EBPβ mRNA (Fig 2B). This indicates that binding of a target RNA by MCPIP1 requires the presence of other MCPIP1 domains. More extensive analysis is needed to elucidate the mechanism of substrate processing by MCPIP1.

**Fig 3:**
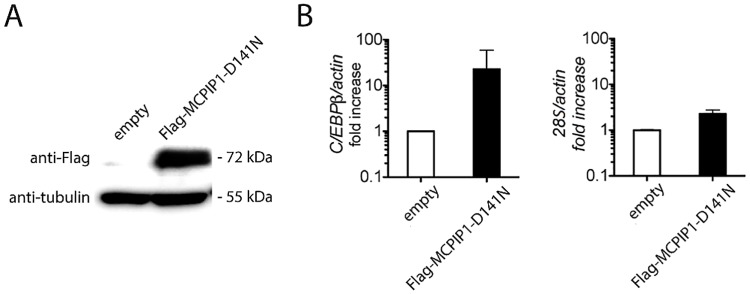
MCPIP1 binds C/EBPβ transcript *in vivo*. HepG2 cells were transfected with an empty plasmid or plasmid coding for enzymatically inactive MCPIP1-D141N with linked Flag tag at N-terminus. Cell lysates were immunoprecipitated with anti-Flag antibodies followed by RNA extraction. **A**. Western blot showing expression of a MCPIP1 and tubulin as a loading control. **B**. Isolated RNA was reverse transcribed and analyzed by the real-time PCR using primers specific to C/EBPβ or 28S RNA with actin specific primers as an internal reference. These are representative results of two independent experiments.

To further confirm an interaction between MCPIP1 and C/EBPβ mRNA, we performed a RIP assay, we performed a RIP assay. To obtain the highest possible amount of isolated RNA, we overexpressed the enzymatically inactive FLAG-MCPIP1-D141N mutant. The presence of MCPIP1-D141N was verified by western blot (Fig 3A). Results of the RIP analysis showed that the FLAG-MCPIP1-D141N precipitate was significantly enriched by a C/EBPβ transcript (Fig 3B). On the other hand, transcript coding for 28S RNA was only slightly increased in Flag-MCPIP-D141N precipitated sample in comparison to control (actin transcript). This demonstrates an *in vivo* interaction between MCPIP1 and the 3'UTR of C/EBPβ mRNA.

### MCPIP1 directly cleaves the 3’UTR of C/EBPβ transcript

**Fig 4:**
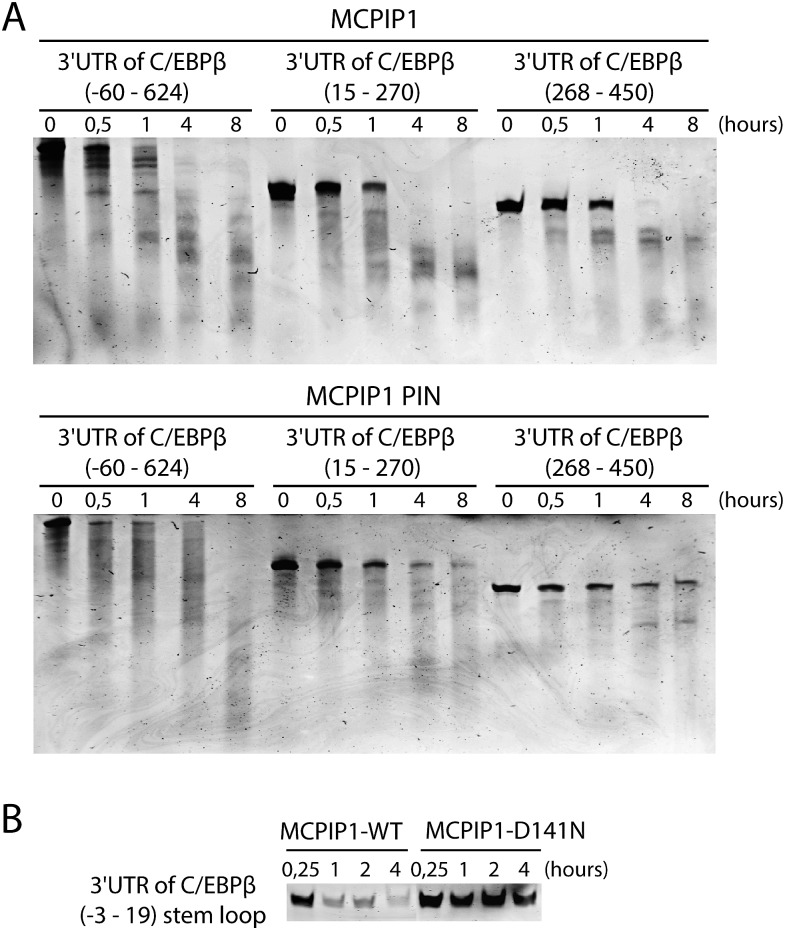
MCPIP1 directly cleaves the 3’UTR of C/EBPβ mRNA. **A**. Cleavage assay of the 3'UTR fragments of C/EBPβ mRNA combined with recombinant MCPIP1 (residues 1–599) or MCPIP1 PIN (residues 134–327). **B**. Cleavage assay of the synthetized 3'UTR of C/EBPβ mRNA (-3-19) stem loop structure combined with recombinant MCPIP1-WT or MCPIP1-D141N as a control.

To clarify whether MCPIP1 binding of the C/EBPβ mRNA 3'UTR results in degradation of CEBPβ transcript we performed a cleavage assay, where recombinant MCPIP1 was mixed and incubated *in vitro* with the 3'UTR fragments of C/EBPβ mRNA for 0.5, 1, 4 and 8 hours. We observed that all analyzed 3'UTR fragments of C/EBPβ mRNA underwent MCPIP1-mediated degradation (Fig 4A). However, the degradation rate of the distant 3'UTR fragment of C/EBPβ mRNA (268–450) was significantly slower than that of the full-length 3'UTR of C/EBPβ mRNA (-60-624). Moreover, the region located between between -3 to 19 nt of the 3’UTR of C/EBPβ mRNA was also confirmed as an important in mRNA degradation assay with recombinant wild type MCPIP1 but not with its mutant form (Fig 4B).

The pattern of bands detected after 0.5 h cleavage of the full-length 3'UTR (-60-624) clearly shows that there are several specific sites of endonucleolytic cleavage within the 3'UTR of C/EBPβ mRNA. For the longer reaction times, we observed fragmentation of the 3’UTR that most likely results from less specific interactions between MCPIP1 and the RNA. This study revealed that there are numerous sites of interaction in the 3'UTR of C/EBPβ mRNA, which might differ in their affinity for MCPIP1.

Since it has been shown that the catalytic domain of MCPIP1 is capable of degrading RNA, we performed an additional cleavage assay for a MCPIP1 PIN domain (Fig 4A). We observed that it was capable of degrading RNA and the final products displayed the same pattern as observed for whole protein, although they appeared after longer reaction times. This demonstrates that the isolated PIN domain of MCPIP1 (residues 134–327) exhibit specificity for transcript degradation but it has significantly lower catalytic activity against the 3'UTR of C/EBPβ mRNA than the full-length MCPIP1 protein.

### Identification of potential hairpins in the 3’UTR of C/EBPβ mRNA

**Fig 5:**
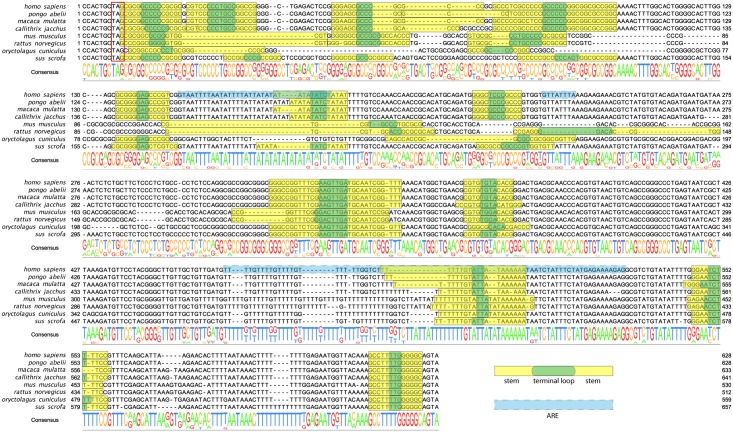
Highly conserved regions of C/EBPβ mRNA might fold into hairpins. Clustal Omega alignment of the C/EBPβ mRNA 3'UTR of eight mammalian species revealed regions of high conservation status. Within these, a set of potential hairpins is marked in yellow and green.

All mRNA identified so far as a targets of MCPIP1 contain a stem-loop in the 3’ UTR [5,7,8]. It has been proven that substrate binding by MCPIP1 requires the presence of a stem-loop within an RNA [7]. To investigate the possibility of MCPIP1 involvement in C/EBPβ mRNA stability, we searched for hairpins in the 3’UTR. Using the *Ensemble* database and ClustalW [20,21] we aligned 3'UTR sequences of C/EBPβ mRNA from 8 mammalian species. The highly evolutionarily conserved regions have been further analyzed for the presence of potential secondary structures using the *mfold* web server [22] (Fig 5). We identified many sites within the 3’UTR of C/EBPβ mRNA that fold into hairpins with a high probability. These regions were spread within the 3’UTR and were appeared to be highly conserved. However, further functional studies are essential to show which stem-loops from the 3’UTR of C/EBPβ mRNA are essential for degradation triggered by MCPIP1.

## Discussion

C/EBPβ belongs to a family of leucine-zipper transcriptions factors. According to the Human Protein Atlas, C/EBPβ is a ubiquitous protein with the highest expression levels in intestine, lungs, skin, adipose tissue, breast, skeletal muscle and liver [23]. Since C/EBPβ activates a broad range of genes essential for homeostasis, including genes related to immune function, metabolism, and growth, the regulation of C/EBPβ activity must be accurate. It has been shown that C/EBPβ activity is controlled on transcriptional and post-translational levels. However, little is known about the post-transcriptional control of C/EBPβ expression. The HuR protein has been shown to bind the C/EBPβ transcript and enhance its stability [24,25], but according to our knowledge no destabilizing agent for the C/EBPβ transcript has been described so far.

MCPIP1 is an endonuclease involved in mRNA decay by selective cleavage of mRNAs within their 3’UTR. We found previously that MCPIP1 overexpression led to a decrease in mouse C/ebpβ mRNA and protein levels. Furthermore, experiments done on HepG2 cells with actinomycin D confirmed that MCPIP1 reduces half-life of human C/EBPβ transcript [17]. Additionally, Yang and co-workers showed that MCPIP1 overexpression reduces the amount of the CREB [26], which is a transcription factor promoting *C/EBPβ* gene expression. Here, we investigated the influence of MCPIP1 on the stability of the C/EBPβ transcript.

After applying an *in vitro* cleavage assay followed by a luciferase-reporter assay and RNA immunoprecipitation (RIP), we demonstrated that MCPIP1 can regulate levels of C/EBPβ through a direct endonucleolytic cleavage of the 3'UTR of the C/EBPβ mRNA. To identify the region of the 3’UTR that might be succeptible for this degradation, we analysed MCPIP1-dependent degradation of for four different parts of the 3’UTR spanning -3-19, 15–270, 268–450, 432–624 nucleotides. We observed that all analysed regions of the 3'UTR C/EBPβ mRNA were cleaved by the full-length recombinant MCPIP1 with similar efficiency. However, a luciferase assay of the distant regions spanning the nucleotides from 268 to 624 did not confirm their susceptibility to degradation. This may result from the low sensitivity of the luciferase assay, since for both tested constructs we observed a slight decrease in luciferase activity, although this decrease was not statistically significant. Nevertheless, this data indicates that the 3’UTR of the human C/EBPβ mRNA contains few sites of interaction with the MCPIP1 endonuclease. Analysis of evolutional conservation followed by the prediction of secondary structures revealed in the 3’UTR of C/EBPβ the presence of many potentially relevant hairpins. Two of these, located in human C/EBPβ 171–185 and 365–376 nt, can be targeted by MCPIP1 as they exhibit a Py-Pu-Py pattern in the loop regions (TAT and TGT, respectively) and either a G or A on the second position in the loop has been shown to be crucial for MCPIP1 suppression [7]. It is likely there are additional MCPIP1 targets in the 3’UTR of C/EBPβ mRNA. We observed that region spanning nucleotides from -3-19 was also cleaved by MCPIP1, both *in vitro* and *in vivo*. According to bioinformatics tools this region may also create a hairpin containing a different than Py-Pu-Py nucleotide pattern in the loop. However, Lu and co-workers showed that targets other than the Py-Pu-Py loops are also susceptible for degradation by MCPIP1 [27]. The impact of this particular hairpin on C/EBPβ mRNA remains uncertain as Mino and co-workers showed that only a distance greater than 20 nt between the hairpin and stop codon assures suppression of MCPIP1 on IL-6 3’UTR [7]. Such conditions were created in the genetic constructs we used, but were not present in the original C/EBPβ mRNA. However, this hairpin does not exhibit evolutionary conservation, as it was not present in C/EBPβ mRNA of analysed rodents. The significance of stem loop structures detected with bioinformatics tools in the 3’UTR of C/EBPβ mRNA in the specific degradation triggered by MCPIP1 require further, more complex studies.

Since it has recently been proposed that cleavage of mRNA is exerted by MCPIP1 dimers [10], we tested whether a fragment of MCPIP1 that is enzymatically active but unable to form oligomers can degrade the 3’UTR of C/EBPβ mRNA *in vitro*. We chose a MCPIP1 fragment consisting of residues 134–327 as both C-terminal and N-terminal fragments have been described as an important for MCPIP1 dimer formation [10,28]. We observed significantly lower efficiency of RNA biding and degradation capacity of analysed MCPIP1 fragments compared to a wild-type protein.

Interestingly, a recently found inductor of MCPIP1 expression is C/EBPα [29]. Considering that C/EBPα and C/EBPβ act cooperatively, one might expect that expression of MCPIP1 depends on C/EBPβ as well. In favour of this hypothesis is the identification of a negative feedback loop between MCPIP1 and its other activator, NF-κB [30].

There are four C/EBPβ transcripts resulting from the alternative positions of the translation start site. Isoforms LAP* and LAP are considered to be transcriptional activators, while isoform LIP is their natural, dominant-negative inhibitor and thus transcriptional repressor [31]. Although mRNAs coding for C/EBPβ isoforms are different in size, their 3’UTRs have the same length, thus are equally susceptible to MCPIP1 cleavage. It is likely that the ratio of LAP*/LIP or LAP/LIP isoforms is more important than the concentration. From this point of view, MCPIP1 might be regarded as regulator of C/EBPβ activity. Transcriptionally active isoforms of C/EBPβ are generally regarded as the key regulators of IL-6 signalling while other MCPIP1 targets, including NF-κB, coordinate signalling of IL-1β. Both transcription factors must act synergistically to activate genes coding for IL-6, IL-8, and IL-12 [15,16]. Interestingly, the mRNA of these proteins is also targeted by MCPIP1 [4,5,18]. Moreover, MCPIP1 has been proven to inhibit NF-κB activity and cleave c-Rel mRNA [30,32–34].

Our previous and present results indicate that MCPIP1 represses expression of C/EBPβ in mouse and humans [17]. This brings new insight into understanding of regulatory networks and orchestrated response to IL-1 and IL-6 cytokines. This also demonstrates various MCPIP1 functions in the process of silencing of inflammatory response. Down-regulation of C/EBPβ by MCPIP1 might be also potentially relevant to clinical applications, since enhanced expression of C/EBPβ exists in the background of pathological conditions such as, cancer and metabolic syndrome [35–37].
